# General anaesthesia for caesarean section in a patient with neuromyelitis optica spectrum disorder (NMOSD)

**DOI:** 10.1016/j.ijscr.2019.03.057

**Published:** 2019-04-05

**Authors:** Sasima Dusitkasem, Pattika Subsoontorn

**Affiliations:** Department of Anesthesiology, Faculty of Medicine Ramathibodi Hospital, Mahidol University, Bangkok, Thailand

**Keywords:** Caesarean section, Demyelinating disease, General anaesthesia, Neuromyelitis optica spectrum disorder (NMOSD)

## Abstract

**Introduction:**

Neuromyelitis optica spectrum disorder (NMOSD) is a rare demyelinating disorder affecting the spinal cord and optic nerves. The anesthetic management in parturient women with NMOSD are controversial and remains challenging.

**Presentation of case:**

A 35-year-old G2P1 woman at 36 weeks of gestation with NMOSD presented for pre-anesthesia assessment prior to caesarean section. Her NMOSD had been diagnosed four years previously and was treated with intravenous methylprednisolone (IVMP) and plasma exchange (PLEX). She underwent general anesthesia without developing adverse respiratory events or signs and symptoms of muscle weakness.

**Discussion:**

Both neuraxial and general anaesthesia for NMOSD have been described in previous case reports. Risk factors of general anesthesia in pregnancy with NMOSD must be weighed against the likely risk of NMOSD relapse and the potential deteriorating neurological symptoms after neuraxial anesthesia.

**Conclusion:**

A multidisciplinary collaboration together with careful anesthetic consideration is required for the anesthetic and perioperative management of these patients.

## Highlights

• We report the anaesthetic management of a patient with pre-existing NMOSD undergoing a caesarean delivery.
• Multidisciplinary collaboration and careful patient counselling were essential to optimize maternal and foetal outcomes.
• The use of neuromuscular monitoring and sugammadex reversal might play an important role in anesthetic management of patients with NMOSD.

## 1 Introduction

Neuromyelitis optica spectrum disorder (NMOSD) is a demyelinating disorder affecting the spinal cord and optic nerves. It is typically characterized by acute attacks of unilateral or bilateral optic neuritis and transverse myelitis. Obstetric patients with NMOSD have an increased risk of miscarriage and higher postpartum relapses especially during the first 3 months postpartum. Few published reports are available regarding the anaesthetic management in parturient women with NMOSD because of the rarity of this disease. Therefore, choices of anaesthetic techniques are highly debated and contradictory. Thus, a multidisciplinary collaboration and careful patient counselling are essential to optimize patient outcome. We report the anaesthetic management of a patient with pre-existing NMOSD undergoing a caesarean delivery at 36 weeks. Our case report was approved by Institutional Ethics Committee (Approval number: 02-61-76; Date of approval: March 7^th^, 2018). The patient has provided “written consent” for this publication. This work has been reported in line with SCARE criteria [1].

## 2 Presentation of case

A 35-year-old G2P1 woman 153 cm tall and weighing 58 kg with NMOSD was referred to our institute for an urgent caesarean delivery at 36 weeks of gestation because of the preterm premature rupture of membranes (PPROM). Her previous pregnancy and labour were uneventful at 27 years of age. Four years later, the patient developed new onset of lower extremity paraesthesia and paraplegia. She was unable to ambulate without assistance and had altered visual acuity in her left eye. NMOSD was diagnosed by means of positive serology testing for anti-aquaporin-4 antibody (AQP4 Ab). Clinical improvement was noted after the patient received 5 cycles of intravenous methylprednisolone (IVMP) and plasma exchange (PLEX). Although the patient experienced 3 relapses, her symptoms were improved with IVMP and PLEX without having significant neurological disabilities. She has remained in remission for the past 2 years and has reported no change in symptoms during the pregnancy. The patient’s medications included oral prednisolone, gabapentin, amitriptyline and prenatal vitamins.

Preoperative neurologic examination revealed normal mental status and normal visual acuity. The patient had motor power grade 4/5 for both lower extremities and diminished sensation to pinprick from the level of T6 to T9. Other physical examination and airway evaluations were unremarkable. Vital signs were within the normal limits. The preoperative laboratory investigation results were within the normal ranges.

We informed the patient of all the risks and benefits of anaesthetic techniques. General anaesthesia was preferred regarding the concern of deteriorating neurological symptoms after neuraxial anaesthesia. Routine non-invasive monitoring was established. The patient's initial blood pressure was 127/62 mmHg, heart rate was 90 beats/min and SpO_2_ was 98% in room air. Neuromuscular monitoring was conducted by using a TOF Watch SX. A baseline TOF ratio of 1.0 was recorded. After preoxygenation, rapid-sequence induction was performed using intravenous propofol 3 mg/kg, lidocaine 1 mg/kg, and rocuronium 1.2 mg/kg. General anaesthesia was maintained with sevoflurane and oxygen. The operation was uneventful. A 3,000-g newborn male was delivered with neonatal Apgar scores of 9 and 10 at 1 and 5 min, respectively. Intravenous carbetocin 100 IU was administered slowly over 1 min after umbilical cord clamping. Intravenous midazolam 5 mg and morphine 10 mg were administered. The estimated total blood loss was 400 ml, which was replaced with 1100 ml of crystalloid.

Deep neuromuscular block (TOF count of 0) was observed during the entire surgery. The time from the intubating dose of rocuronium administration to the appearance of the first response (TOF count of 1) was approximately 60 min. Neuromuscular blockade was reversed with sugammade x 4 mg/kg. Tracheal extubation was performed after the patient was awake and responsive with a TOF ratio of 0.94. Then, the patient was transferred to the intensive care unit (ICU) for clinical observation for the first 24 h after surgery. Her postpartum period was uneventful. She did not exhibit adverse respiratory events or develop signs and symptoms of muscle weakness. Pain was controlled with an intravenous morphine patient-controlled analgesic (PCA) pump for 1 day. The patient was followed closely by a neurologist. She was discharged along with a healthy neonate two days later without evidence of NMOSD relapse. Rituximab therapy was initiated three weeks postpartum for relapse prevention. At 3 and 6 months follow-up by telephone call, she reported no worsening of her neurological symptoms and her neurologic examination from her medical records showed no evidence of disease exacerbation.

## 3 Discussion

Neuromyelitis optica spectrum disorder (NMOSD) is a rare demyelinating disease of the central nervous system (CNS) that predominantly affects the optic nerves and spinal cord [2]. Following the discovery of serum anti-aquaporin-4 IgG (AQP4-IgG), a specific diagnosis could be made in the presence of this biomarker. Experiments have shown that anti-aquaporin-4 IgG may play a role in the pathogenesis of NMOSD and can be used to differentiate NMOSD from other demyelinating syndromes such as multiple sclerosis [3,4].

Epidemiology studies have recognized gender and ethnicity as important variables in the prevalence of NMOSD. In Asian countries, the prevalence ranges from 2.6 to 3.65 per 100,000 in India and Japan, respectively, which is higher compared with that for Caucasians [5,6]. NMOSD predominantly affects women and often presents during childbearing years [7]. The interactions between pregnancy and NMOSD are not well established. Some studies reported a higher risk of miscarriage, pre-eclampsia, pregnancy-related attacks of NMOSD, and higher postpartum relapses, especially during the first 3 months postpartum [[8], [9], [10]].

Literature on the anesthetic management in NMOSD obstetric patients is limited and yields conflicting results. Providing neuraxial anesthesia for these patients is controversial. Pre-existing demyelinated neurons may be more susceptible to local anaesthetic (LA) neurotoxicity. Two case reports described disease exacerbation following neuraxial anaesthesia in one patient undergoing minor orthopaedic surgery and the other undergoing caesarean section [11,12]. In contrast, an uneventful postoperative outcome has been demonstrated in a neuromyelitis optica (NMO) patient who received epidural anaesthesia for an urgent caesarean delivery related to a non-reassuring foetal heart tracing at 37 weeks gestation [13]. Furthermore, a retrospective cohort of 20 NMO obstetric patients found that epidural analgesia did not have an aggravating effect on the disease [9]. On the basis of available knowledge, it seems to be a temporal consequence rather than a cause–effect relationship; however, it could be a confounding factor for the early diagnosis of disease exacerbation [12].

Risk factors of general anesthesia in pregnancy with NMOSD must be weighed against the likely risk of NMOSD relapse and the potential deteriorating neurological symptoms after neuraxial anesthesia. According to the available literature, there were some concerns about increased neuromuscular junction (nmj) responses to muscle relaxants in a patient with NMOSD. A case report demonstrated incomplete recovery from neuromuscular blockade with rocuronium after extubation in a patient with NMO who underwent caesarean delivery under general anaesthesia. The patient was complicated by respiratory weakness responsive to bimodal positive airway pressure (BiPAP) despite having an additional dose of neostigmine (total dose 0.1 mg/kg) for neuromuscular reversal [14].

The decision to proceed with general anesthesia was made after a comprehensive neurological evaluation and discussion about the risks and benefits of each anesthetic techniques with the patient. Due to the concern of increase sensitivity to muscle relaxant, neuromuscular monitoring was observed during the surgery. However, we did not observe prolonged neuromuscular blockade in our patient. A TOF count of 1 reappeared approximately 60 min following the intubating dose of rocuronium administration and a TOF ratio of 0.94 was recorded shortly after sugammadex reversal. Moreover, she did not exhibit adverse respiratory events or develop signs and symptoms of muscle weakness during the hospital stay. The patient was followed closely by a neurologist because of the high probability of disease exacerbation in the postpartum period. She reported no worsening of her neurological symptoms and her neurologic examination showed no evidence of disease exacerbation.

## 4 Conclusion

We report the anaesthetic management of a patient with pre-existing NMOSD undergoing a caesarean delivery. Multidisciplinary collaboration and careful patient counselling were essential to optimize maternal and foetal outcomes. With respect to general anesthesia, neuromuscular monitoring and the use of sugammadex reversal might play a role regarding the concern of abnormal neuromuscular junction (nmj) response to muscle relaxant in a patient with NMOSD. Further reports and multicentre studies are necessary for a better understanding of the influence of NMOSD on anaesthesia to determine the appropriate anaesthetic management for parturient women with this condition.

## Conflicts of interest

None.

## Sources of funding

None.

## Ethical approval

This case report was approved by the Faculty of Medicine Ramathibodi Hospital Ethics Committee (Approval number: 02-61-76; Date of approval: March 7th, 2018) with the document of approval attached.

## Consent

The patient has provided “written consent” for this publication.

## Author contribution

Sasima Dusitkasem MD:

This author drafted the manuscript, conceived of and critically revised the final draft for important intellectual content

Pattika Subsoontorn MD:

This author helped drafting the manuscript.

All authors read and approved the final version of the manuscript.

## Registration of research studies

NA.

## Guarantor

Sasima Dusitkasem MD.

## Provenance and peer review

Not commissioned, externally peer-reviewed
